# *Trichosanthes metamorpha* (Cucurbitaceae), a striking new species from Guangxi, China

**DOI:** 10.3897/phytokeys.280.190832

**Published:** 2026-10-05

**Authors:** Ruo-Zhou Wang, Zhao-Hui Ran, Hanno Schaefer, Sheng-Wei Wang, Neng Wei, Qing-Feng Wang

**Affiliations:** 1 State Key Laboratory of Plant Diversity and Specialty Crops, Wuhan Botanical Garden, Chinese Academy of Sciences, Wuhan 430074, Hubei, China State Key Laboratory of Plant Diversity and Specialty Crops, Wuhan Botanical Garden, Chinese Academy of Sciences Wuhan China; 2 College of Horticulture and Gardening, Heilongjiang Bayi Agricultural University, Daqing 163319, Heilongjiang, China College of Horticulture and Gardening, Heilongjiang Bayi Agricultural University Daqing China; 3 University of Chinese Academy of Sciences, Beijing 100049, China University of Chinese Academy of Sciences Beijing China; 4 Plant Biodiversity, Department of Life Science Systems, Technical University of Munich (TUM), Emil-Ramann-Str. 2, D-85354 Freising, Germany Plant Biodiversity, Department of Life Science Systems, Technical University of Munich (TUM) Freising Germany; 5 Sino-Africa Joint Research Center (SAJOREC), Chinese Academy of Sciences, Wuhan 430074, Hubei, China Sino-Africa Joint Research Center (SAJOREC), Chinese Academy of Sciences Wuhan China

**Keywords:** Morphology, new species, phylogeny, taxonomy, *
Trichosanthes
*

## Abstract

*Trichosanthes metamorpha*, a new species from Guangxi Autonomous Region, China, is described and illustrated. Morphologically, it closely resembles *T. dunniana* and *T. pubera* but can be readily distinguished by its tiny black dots about the size of a pinpoint (c. 0.1 mm in diameter) on the stem surface, the leaf shape and basal leaf marking patterns, short and brittle petal fringes, orange-yellow fruit, and broadly ellipsoid seeds without ridges or ornaments. Molecular phylogenetic analyses based on a plastid coding sequence dataset comprising 67 protein-coding genes (cp-CDS) and a concatenated three-locus dataset (ITS, *matK*, and *rpl20-rps12*) consistently place *T. metamorpha* within *T.* sect. *Involucraria*. The integrated evidence of its morphological characteristics and phylogenetic placement supports the recognition of *T. metamorpha* as a distinct species.

## Introduction

*Trichosanthes* Linnaeus (1753: 1008) is one of the most species-rich genera in Cucurbitaceae, comprising c. 100 species and occurring widely from India and Sri Lanka through China, Japan, and Southeast Asia to Papua New Guinea, northern Australia, and the Pacific islands (de Wilde and Duyfjes 2010; de Boer et al. 2012; Nong et al. 2015; Lin et al. 2025). *Trichosanthes* is placed in subfamily Cucurbitoideae, tribe Sicyeae, and subtribe Trichosanthinae (Kocyan et al. 2007). The genus is characterized by petals with conspicuously fringed margins, the presence of a probract at the petiole base in most species, and straight anther thecae. In China, *Trichosanthes* comprises c. 40 species, predominantly distributed in the southern and southwestern regions (Nong et al. 2015). Several species of *Trichosanthes* have long been used in traditional medicine, with *T. kirilowii* Maxim. (1859: 482) being the most widely utilized.

The accumulation of names and extensive morphological variation has rendered *Trichosanthes* a taxonomically “challenging group,” leading to repeated adjustments to generic circumscription and infrageneric delimitation (Huang and Jeffrey 2011; de Boer and Thulin 2012; Lin et al. 2025). Yueh and Cheng (1980) divided *Trichosanthes* into two subgenera and seven sections based on probracts, leaf indumentum, pulp color, and seed morphology. Huang et al. (1997), based on pollen morphology, further supported the sectional framework established by Yueh and Cheng (1980) and proposed the subdivision of sect. *Foliobracteola* into two subsections. Rugayah and de Wilde (1999) proposed the new sect. *Edulis* Rugayah and recognized five sections of *Trichosanthes* in Malesia, emphasizing seed and fruit characters. Using multilocus molecular data, de Boer and Thulin (2012) merged *Gymnopetalum* into *Trichosanthes* for monophyly and erected an infrageneric framework with two subgenera and eleven sections. Recently, large-scale analyses based on whole-plastome data further suggested that *Trichosanthes* may be paraphyletic, with multiple genera nested within it, indicating that current generic boundaries and infrageneric classification warrant further evaluation and revision with more comprehensive sampling and integrated evidence (Lin et al. 2025). However, although Lin et al. sampled broadly across Cucurbitaceae, they covered only nine of the eleven sections recognized by de Boer and Thulin (2012), and species-level representation of the genus remained incomplete. Therefore, their inferences regarding generic circumscription and infrageneric groupings require validation through expanded sampling.

In July 2024, during field investigations in Yangshuo County, Guangxi Zhuang Autonomous Region, China, an unusual *Trichosanthes* plant with conspicuous white patterns on the adaxial leaf surface was discovered, and a small number of living specimens were collected for cultivation in the greenhouse of Wuhan Botanical Garden, Chinese Academy of Sciences. When the type locality was revisited in July 2025, the plant, at anthesis, was observed to bear conspicuously enlarged deep-red bracts and a pinkish apricot-colored corolla. Its overall morphology resembles that of the geographically proximate *Trichosanthes dunniana* H. Lév. (1911: 148) and *T. pubera* Blume (1826: 936) in some characters, but it differs consistently in stem, leaf surface, and fruit color, most notably in having extremely short dentate petal fringes that are incurled and relatively thick-textured yet brittle. Based on these observations, it was preliminarily considered to belong to *Trichosanthes*. To clarify its taxonomic status, detailed morphological comparisons and molecular phylogenetic analyses were conducted; the combined evidence supports the recognition of the Yangshuo population as a new species of *Trichosanthes*. The new species is formally described and illustrated below.

## Material and methods

### Morphology comparison

The dried specimens of the new species were collected from Yangshuo County, Guangxi Province. In addition, photographs documenting the habitat and plant habit were taken during field surveys. Morphological features were observed and photographed using Sony NEX-5N, DSC-HX30, and Nikon D810 FX cameras, and measurements were taken using ImageJ software (https://imagej.net/ij/). For morphological comparison, type specimens and a large number of non-type specimens of closely related species were consulted, either by examining specimens on site at herbaria (HIB, K, and PE; herbarium acronyms follow Thiers (2026)) or by examining digitized specimens online, including the Chinese Virtual Herbarium (www.cvh.ac.cn/), Global Plants on JSTOR (https://plants.jstor.org/), and the Global Biodiversity Information Facility (https://www.gbif.org/). Voucher specimens were deposited in the herbarium of Wuhan Botanical Garden, CAS (HIB), and the herbarium of the Institute of Botany, CAS (PE).

### DNA sequencing and plastome/nrDNA assembly

Leaf material of the proposed new species was collected in the field and dried in silica gel. Total genomic DNA was extracted from the silica-dried leaves using the Mag-MK kit (Sangon Biotech, Shanghai, China). DNA integrity was assessed by 1% agarose gel electrophoresis, and DNA concentrations were quantified using a Qubit 2.0 fluorometer (Thermo Fisher Scientific, USA). Libraries were prepared using the NEBNext Ultra II DNA Library Prep Kit (New England Biolabs, USA) and sequenced as paired-end reads on the Illumina HiSeq 2000 platform at BGI (Shenzhen, China). Plastome assembly was conducted using GetOrganelle v. 1.7.4 (Jin et al. 2020), with *T. pubera* subsp. *rubriflos* (MZ427952) as the reference sequence. All parameters were set to their defaults, except for the number of iterations, which was set to 25. Annotation was performed using GeSeq v. 2.03 (Tillich et al. 2017) and manually reviewed in Geneious Prime v. 2025.0.2. Ambiguous protein-coding genes were further verified using BLASTn (Altschul et al. 1990). Nuclear ribosomal DNA (nrDNA) sequences were assembled using the custom workflow in GetOrganelle (default settings) and manually reviewed in Geneious Prime v. 2025.0.2.

### Phylogenetic analyses

To determine the phylogenetic relationships of *Trichosanthes metamorpha*, *Luffa* Miller (1754: 806), *Hodgsonia* Hook.f. & Thomson (1854: 257), and *Sicyos* Linnaeus (1753: 1013) were used as outgroups, following the methodology outlined by de Boer et al. (2012) and Lin et al. (2025). Sequences for the nuclear ribosomal internal transcribed spacer (ITS), *matK*, and *rpl20-rps12* regions were obtained from GenBank. The concatenated three-locus dataset (ITS, *matK*, and *rpl20-rps12*) comprised 72 accessions, represented by 66 ITS, 60 *matK*, and 63 *rpl20-rps12* sequences. In addition, 38 publicly available complete plastomes of Sicyeae, including 32 accessions of *Trichosanthes*, were downloaded from GenBank based primarily on the sampling of Lin et al. (2025). Together with the newly generated plastome of *T. metamorpha*, the plastid coding sequence (cp-CDS) dataset comprised 39 accessions. Finally, two datasets were constructed: (1) a concatenated three-locus dataset (ITS, *matK*, and *rpl20-rps12*) and (2) a plastid coding sequence dataset comprising 67 protein-coding genes shared among all sampled plastomes (cp-CDS dataset). These two datasets are widely used in studies of this genus (Liu et al. 2021; Lin et al. 2025). Sample voucher information and GenBank accession numbers are provided in Suppl. material 1.

Sequence alignment was performed using MAFFT v. 7.487 (Katoh and Standley 2013). The optimal partitioning scheme and substitution models were selected using PartitionFinder 2 (Lanfear et al. 2017). Maximum likelihood (ML) analysis was conducted using IQ-TREE v. 1.6.8 (Nguyen et al. 2015), and Bayesian inference (BI) analysis was performed using MrBayes v. 3.2.6 (Ronquist et al. 2012). To assess potential incongruence between the nuclear ITS partition and the plastid markers, an incongruence length difference (ILD) test was performed using PAUP v. 4.0b10 (Swofford 2002), as described by Farris et al. (1995). Finally, the phylogenetic tree was customized using the online tool iTOL v. 5 (Letunic and Bork 2021).

## Results

### Morphological comparison

*Trichosanthes metamorpha* is most similar to *T. dunniana* and *T. pubera* (see Table 1). All three species have papery, palmately lobed leaves and inflorescences that are reddish to dark red overall, but *T. metamorpha* can be distinguished from the other two by the following combination of characters: its stem is glabrous and marked with tiny black dots, densest at the nodes, whereas *T. dunniana* has stems with punctations and hairy nodes, and *T. pubera* has hairy stems, including at the nodes. In *T. metamorpha*, the basal leaves bear conspicuous white patterns, which have not been documented in the available specimens, descriptions, or field photographs of *T. dunniana* and *T. pubera* examined in this study. The petals of *T. metamorpha* bear very short, dentate fringes that are incurled and relatively thick and brittle (2.0–2.5 mm long); in contrast, both *T. dunniana* and *T. pubera* have longer, filiform, flat, and flimsy fringes (8–12 mm long). Seeds of *T. metamorpha* are broadly elliptic, pale brownish gray, smooth, and lack lateral ridges; those of *T. pubera* are elongate-elliptic, black or blackish brown, without lateral ridges, and usually have concave ornaments on their surfaces, whereas those of *T. dunniana* are broadly elliptic and pale brownish gray but have shallow elliptic depressions and distinct lateral ridges. In addition, *T. metamorpha* exhibits pronounced ontogenetic variation in leaf morphology: the basal leaves are mostly 3-lobed with white patterns, whereas the apical or youngest leaves are usually deeply 5-lobed and lack patterns. This coordinated change in the degree of lobing and variegation along the stem can serve as auxiliary diagnostic characters.

**Table 1.** Comparison of *Trichosanthes metamorpha*, *T. dunniana*, and *T. pubera*.

**Character**	***Trichosanthes metamorpha***	***Trichosanthes dunniana***	***Trichosanthes pubera***
Stem	Totally glabrous, tiny black dots densest at nodes	Punctate and hairy at nodes	Completely hairy
Leaf shape	Suborbicular; palmately 3–5-lobed	Suborbicular; palmately 5–7-lobed	Broadly ovate to suborbicular; palmately 3–7-lobed
Adaxial leaf surface/patterning	Basal leaves dark glaucous green with conspicuous white patterned, initially pubescent, then glabrescent	Green, pubescent/scabrid; no comparable white pattern documented in the material examined	Dark green, pubescent. No comparable white pattern documented in the material examined
Tendrils	3-branched, glabrous	2–3-branched, punctate	3–5-branched, sparsely hairy
Flower texture	Thick and brittle	Flat and flimsy	Flat and flimsy
Flower fringes	Dentate, incurled, 2–2.5 mm long	Filiform, unfolded, 8–12 mm long	Filiform, unfolded, 8–10 mm long
Fruit	Ovoid, orange-yellow	Narrowly ellipsoid, red	Globose, red
Seed shape	Broadly ellipsoid, slightly swollen; without lateral ridges	Broadly ellipsoid, swollen; with an elliptical pattern and lateral ridges	Elongate-ellipsoid, flat; usually concavely ornamented on surfaces, without lateral ridges
Seed size	c. 15–19 × 9–12 mm, 4–5 mm thick	c. 12 × 8 mm, 3–4 mm thick	c. 12 × 4–5 mm, 1–3 mm thick

### Phylogenetic analysis

**Figure 1.**
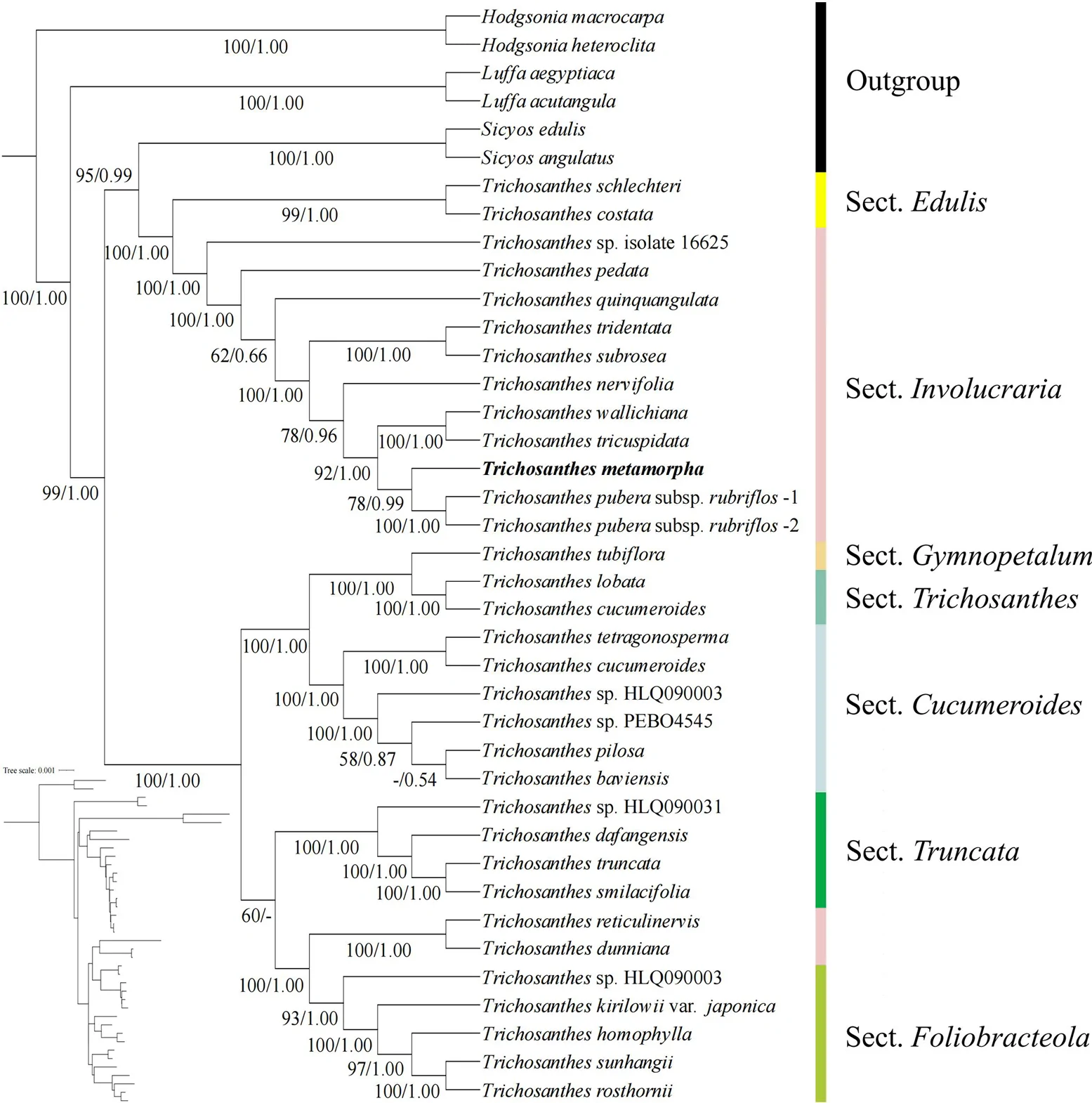
Maximum-likelihood phylogeny of *Trichosanthes* inferred from the cp-CDS dataset comprising 67 plastid protein-coding genes. Maximum-likelihood (ML) and Bayesian inference (BI) analyses recovered identical topologies. Values at nodes indicate ML bootstrap support (BS) and Bayesian posterior probability (PP), respectively. Only BS ≥ 50% and PP ≥ 0.50 are shown, and a dash (-) indicates support below the corresponding threshold. The new species *Trichosanthes metamorpha* is shown in bold.

**Figure 2.**
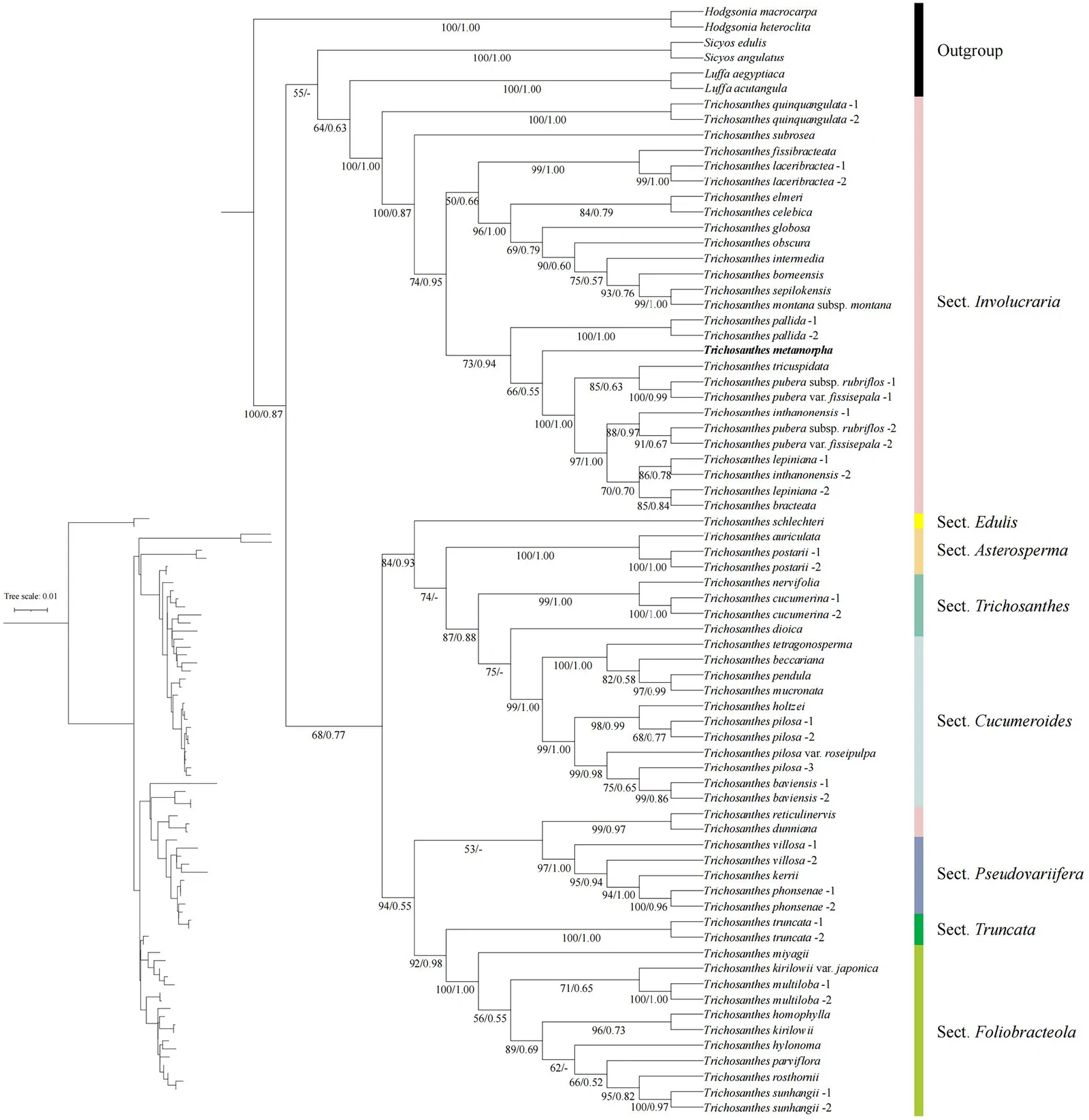
Maximum-likelihood phylogeny of *Trichosanthes* inferred from the concatenated three-locus dataset (ITS, *matK*, and *rpl20-rps12*). Values at nodes indicate ML bootstrap support (BS) and Bayesian posterior probability (PP), respectively. Only BS ≥ 50% and PP ≥ 0.50 are shown; a dash (-) indicates support below the corresponding threshold or the absence of the corresponding clade in the BI topology. The new species *T. metamorpha* is shown in bold.

The cp-CDS dataset comprised 39 accessions and 57,566 aligned positions, of which 51,701 sites (89.8%) were identical, with an overall pairwise sequence identity of 99.1%. The concatenated three-locus dataset (ITS, *matK*, and *rpl20-rps12*) comprised 72 accessions and 2,177 aligned positions, of which 1,139 sites (52.3%) were identical, with an overall pairwise sequence identity of 95.3%. The ILD test detected no significant incongruence between the nuclear ITS and plastid partitions (*P* = 0.012; *P* > 0.01). For the cp-CDS dataset, ML and BI analyses recovered identical topologies (Fig. 1). *T. metamorpha* was recovered as sister to *T. pubera* subsp. *rubriflos* (BS = 78%; PP = 0.99) within *T.* sect. *Involucraria*. *T. dunniana* and *T. reticulinervis* formed a strongly supported clade (BS = 100%; PP = 1.00) outside this lineage and were recovered as sister to sect. *Foliobracteola* (BS = 100%; PP = 1.00). For the concatenated three-locus dataset (ITS, *matK*, and *rpl20-rps12*), the ML and BI analyses showed minor topological differences at several internal nodes. The ML topology is presented in Fig. 2, with Bayesian posterior probabilities mapped onto the corresponding nodes also recovered in the BI analysis. *T. metamorpha* was recovered within *T.* sect. *Involucraria* and as sister to a clade comprising *T. tricuspidata*, *T. pubera* subsp. *rubriflos*, *T. pubera* var. *fissisepala*, *T. inthanonensis*, *T. lepiniana*, and *T. bracteata* (BS = 66%; PP = 0.55). In contrast to their sister relationship with *T.* sect. *Foliobracteola* in the phylogeny based on the cp-CDS dataset, *T. dunniana* and *T. reticulinervis* formed a strongly supported clade (BS = 99%; PP = 0.97) that was sister to T. sect. Pseudovariifera with weak support (BS = 53%).

### Taxonomic treatment

#### Trichosanthes metamorpha

Taxon classification

Plantae

CucurbitalesCucurbitaceae

R.Z.Wang, N.Wei & Q.F.Wang sp. nov.

DA89CF1E-2BE6-5BF7-B144-180063A1ABDF

urn:lsid:ipni.org:names:77407421-1

Figs 3, 4, 5, 6

##### Chinese name.

异叶栝楼.

**Figure 3.**
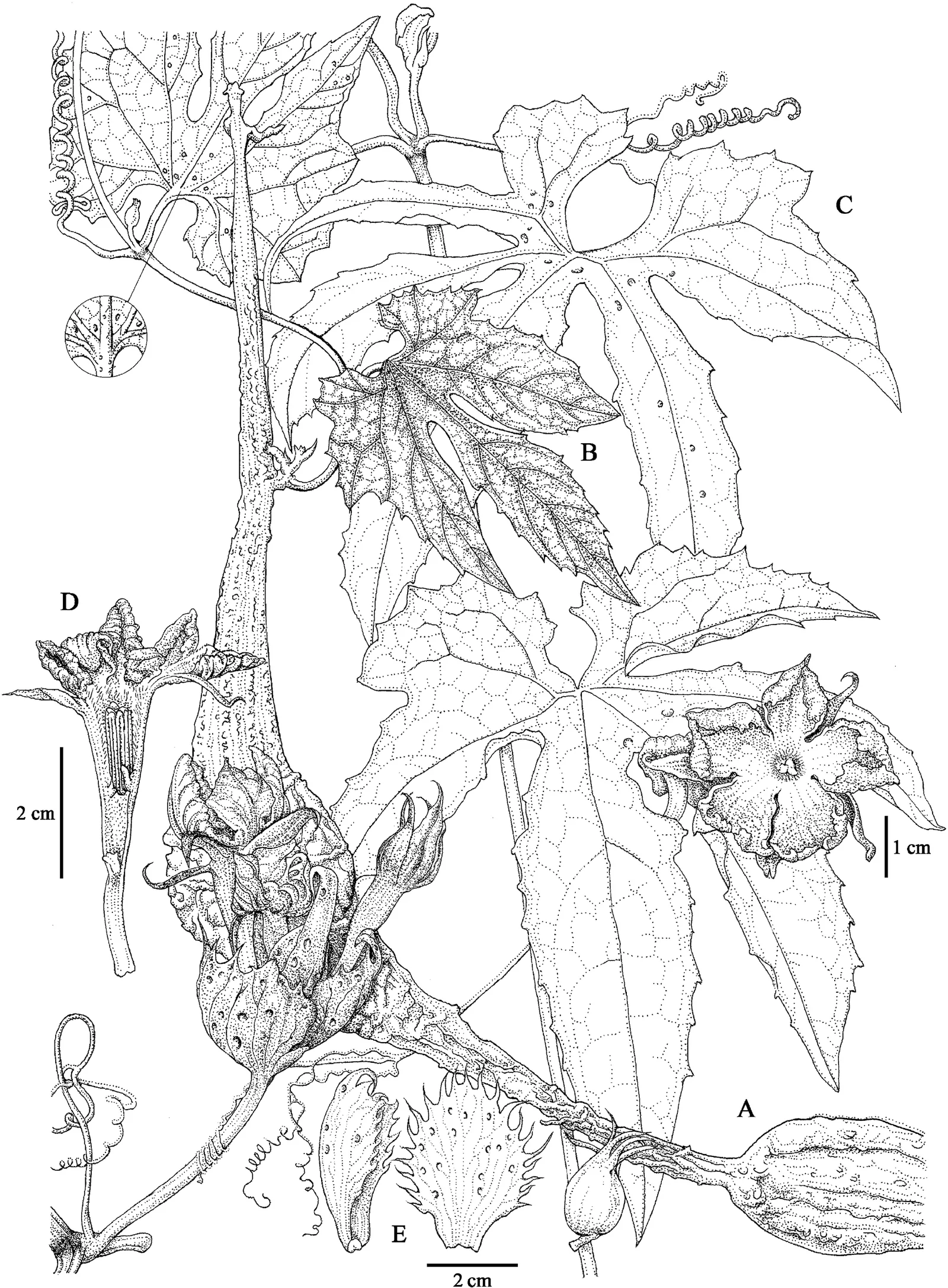
Male plant of *Trichosanthes metamorpha*. **A**. Young tuber; **B**. Basal leaf with detail; **C**. Apical leaf, showing glands; **D**. Opened flower, showing stamens; **E**. Bracts, side and face views. Drawn by Victor Wong.

**Figure 4.**
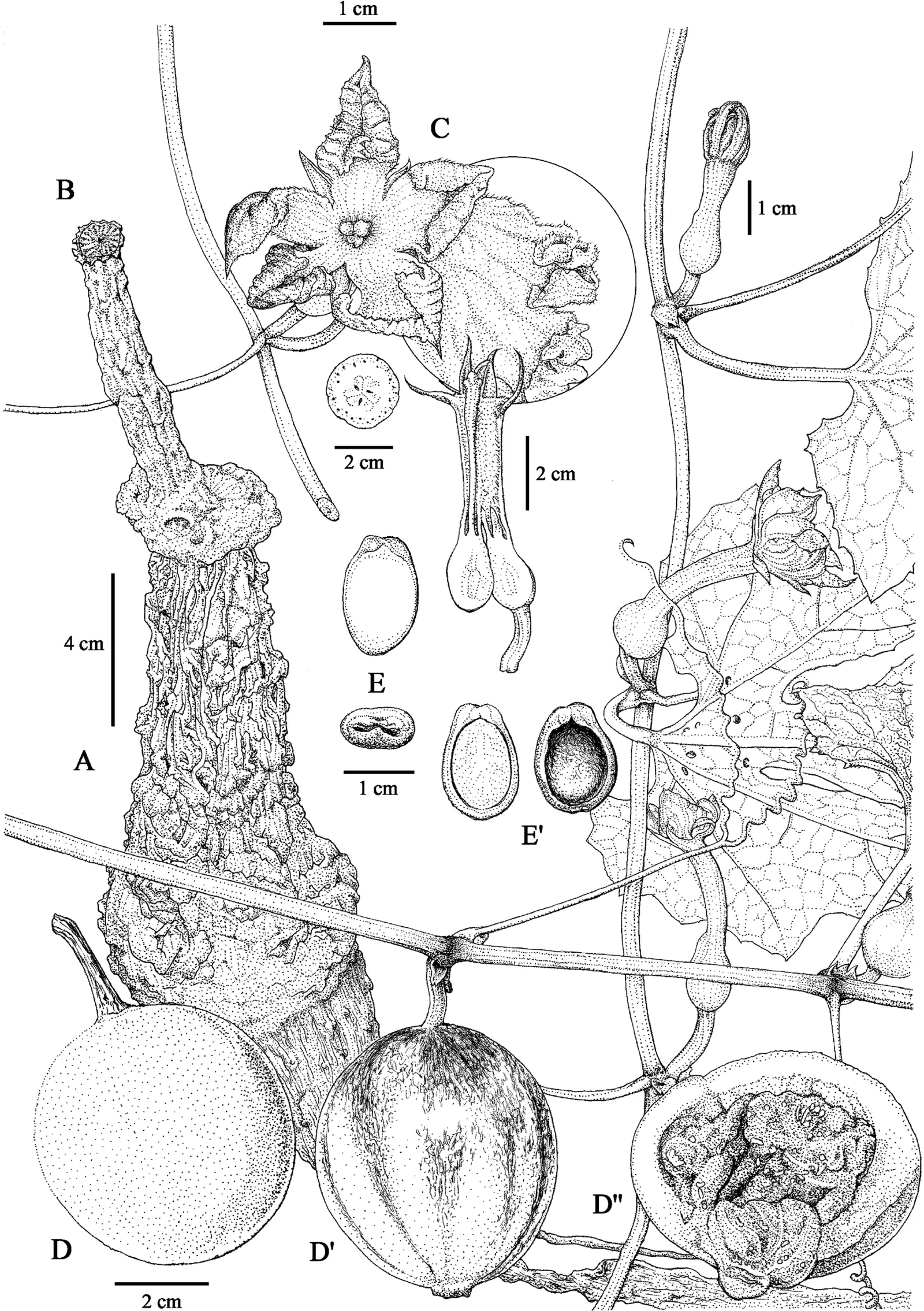
Female plant of *T. metamorpha*. **A**. Tuber; **B**. Detail of climbing stem; **C**. Female flower, top view, with detail of petal; **D**. Mature fruit; **D'**. Immature fruit; **D"**. Opened mature fruit; **E**. Seed, lateral and apical views; **E'**. Opened seed showing internal structure. Drawn by Victor Wong.

**Figure 5.**
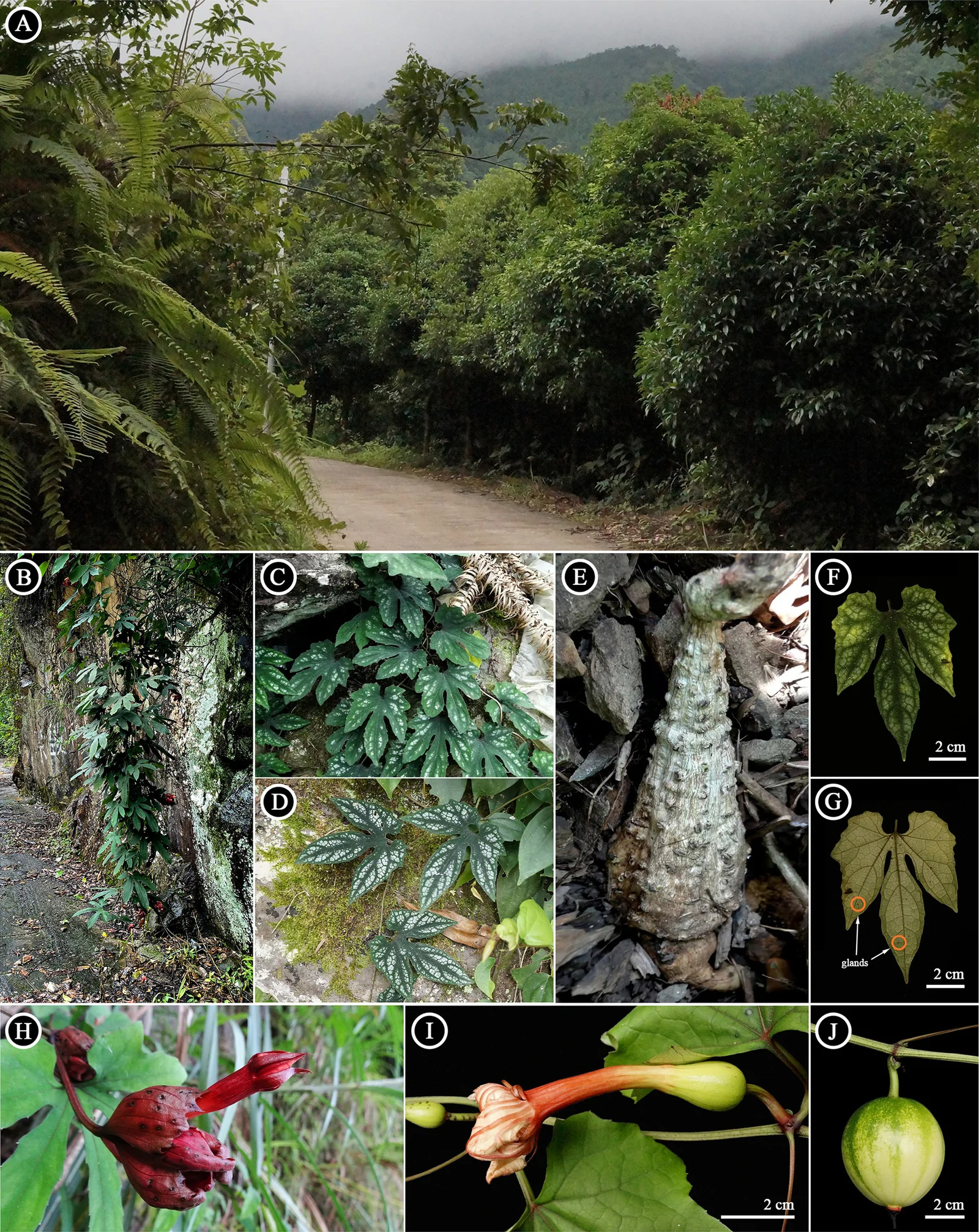
Habitat and morphology of *T. metamorpha*. **A**. Habitat; **B**. Apical leaves; **C**. Medial leaves; **D**. Basal leaves showing white patterns; **E**. Tuber; **F**. Adaxial view of basal leaf; **G**. Abaxial view of basal leaf; **H**. Male flower; **I**. Female flower; **J**. Young fruit, showing green stripes. Photos by Ruozhou Wang.

**Figure 6.**
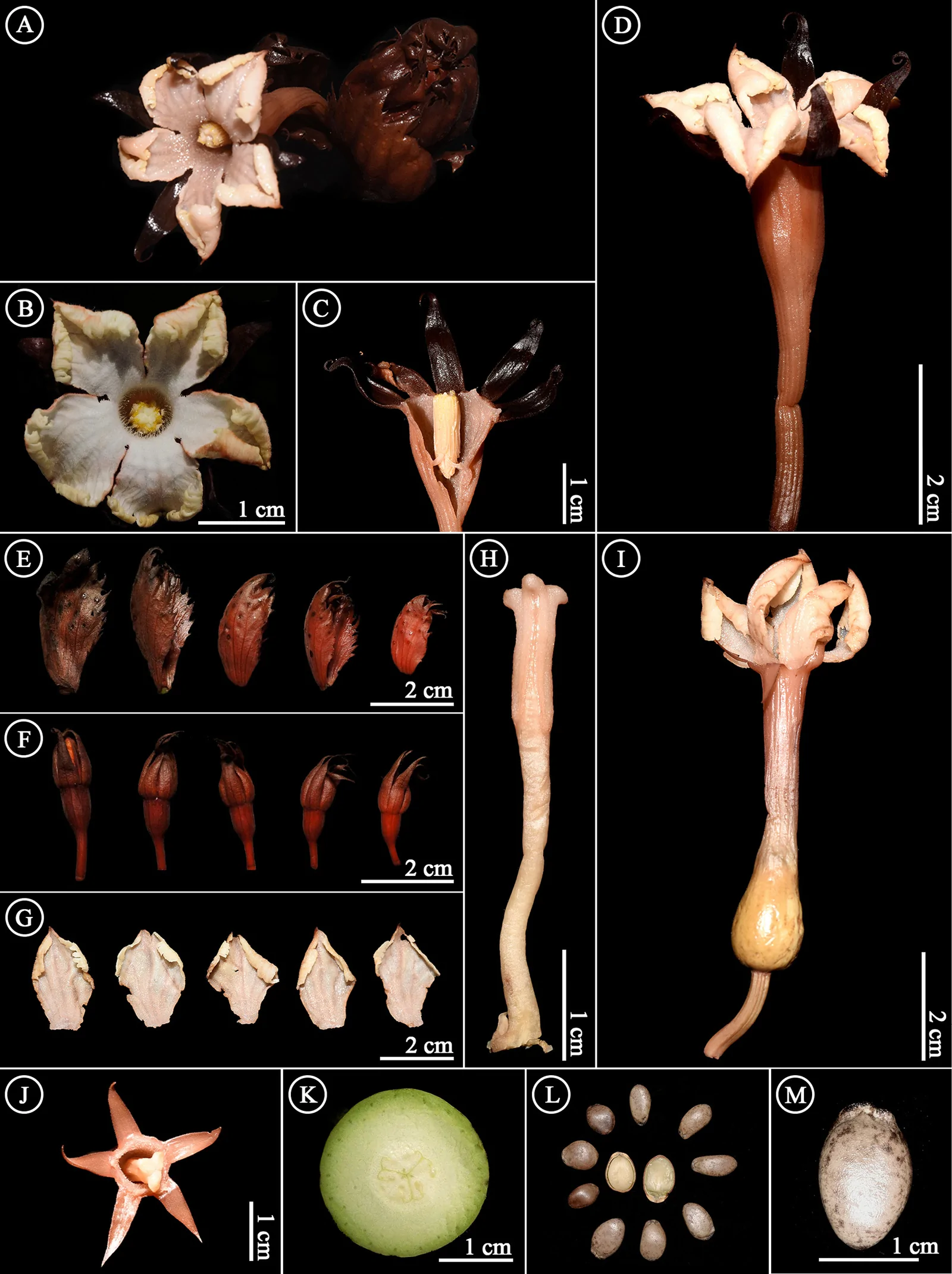
*Trichosanthes metamorpha*. **A**. Male inflorescence and male flower; **B**. Idem, top view, showing incurled petal fringes; **C**. Idem, opened; **D**. Idem, lateral view; **E**. Bracts; **F**. Young male flowers; **G**. Petals of male flower; **H**. Pistil; **I**. Lateral view of female flower; **J**. Calyx and stigma; **K**. Cross-section of young fruit; **L**. Seed; **M**. Close-up of seed. **A, C, D, G–J, L, M**. Photos by Zhaohui Ran; **B, E, F, K**. Photos by Ruozhou Wang.

##### Type.

China • Guangxi: Guilin City, Yangshuo County, elev. 650 m, 24.9837, 110.5874, on moist rock walls by the roadside, 12 Jul. 2025, *R.Z. Wang 1* (holotype: HIB!; isotypes: HIB!, PE!).

##### Additional specimens examined (paratypes).

China • Guangxi: Guilin City, Yangshuo County, Shihuiyao, elev. 910 m, 24.9931°N, 110.6033°E, 12 Jul. 2025, *R.Z. Wang 2* (HIB!); China • Guangxi: Guilin City, Yangshuo County, elev. 650 m, 25.0186°N, 110.5880°E, Chahualing, 12 Jul. 2025, *R.Z. Wang 3* (K!).

##### Diagnosis.

*Trichosanthes metamorpha* is distinct from *T. dunniana* and *T. pubera* by its glabrous stem with tiny black dots (vs. grooved and punctate in *T. dunniana* and pubescent in *T. pubera*), by its basal leaves conspicuously white patterned (vs. not white patterned in both *T. pubera* and *T. dunniana*), and petal margins bearing short, thick, brittle, and dentate fringes (vs. the long, filiform, flat and flimsy fringes in *T. dunniana* and *T. pubera*), by its orange-yellow fruits at maturity (vs. red or orange-red in *T. pubera* and red in *T. dunniana*), and by its smooth seeds (vs. laterally ridged in *T. dunniana* and usually concavely ornamented in *T. pubera*) (Table 1).

##### Description.

***Dioecious*** perennial climber, 3–5 m long or longer. ***Roots*** tuberous. ***Stems*** subterete, ridged and longitudinally sulcate, glabrous; basal stem dark green, upper stem green with tiny black dots, densest at the nodes; old stems whitish, with a corky periderm. ***Probracts*** concave, 3–5 × 5–7 mm, subentire, 2–5 glands present. ***Leaves*** alternate; petiole 4–8 cm long, ridged and longitudinally sulcate, occasionally minutely puberulent. Leaf blade papery, surface scabrous; adaxial surface shortly pubescent when young, becoming glabrescent at maturity; abaxial venation raised, sparsely short-pubescent along veins. Basal leaves oblong, usually deeply 3-lobed, or 5-lobed with the three main lobes deeply divided and the two outermost lobes shallow and inconspicuous, 8–10 × 7–8 cm; apical leaves suborbicular, deeply 5-lobed with the two outermost lobes also deeply lobed, 15–18 × 18–20 cm; lateral lobes triangular, inner lobes oblong; margin finely denticulate; apex acute; base cordate, sinus 1–1.5 cm deep and 1.5–2 cm wide; adaxial surface of basal leaves dark glaucous green with reflective white patterns; adaxial surface of apical leaves green without white patterns but with several scattered glands; abaxial surface of both leaf types pale green, with scattered glands. ***Tendrils*** usually 3-branched (with two slender lateral branches and a stout main branch), up to 20 cm long, glabrous. ***Male inflorescences*** racemose, axillary, 8–12-flowered; peduncle 8–10 cm long, green. ***Male flowers***: Bracts elliptic or obovate, 4–5 × 2–3 cm, dark red, margin sharply dentate, minutely puberulent; abaxial surface with dark, sunken glandular dots; pedicels 4–5 cm long, pale reddish; sepals five, lanceolate, 12–15 × c. 3 mm, dark red, margin entire; calyx tube 5–6 cm long, dark red, outer surface densely villous; corolla pinkish apricot; petals five, obovate, 25–30 × 18–20 mm, with dark red venation abaxially; margins with incurled, dentate fringes; fringe segments truncate at the apex, 2–2.5 × 1–2 mm, texture brittle and thickened; filaments three, anthers synantherous, cylindrical, 13–14 mm long, c. 4 mm in diameter, yellow. ***Female flowers*** solitary; calyx and corolla similar to those of male flowers; ovary pale green, narrowly ellipsoid, 2.0–2.5 × 1.2–1.5 cm; calyx tube 4–5 cm long, red; stigma 3-lobed, pale orange. ***Fruit*** ovoid, 9–10 × 6–7 cm, glabrous; immature fruit pale green with broad green longitudinal stripes, becoming orange-yellow at maturity; pulp dark green; seeds 25–35 per fruit. ***Seed*** broadly ellipsoid, 15–19 mm long, 9–12 mm broad, 4–5 mm thick, pale brownish gray, smooth, without lateral ridges.

##### Phenology.

Flowering from July to August. Fruiting from July to October.

##### Distribution and habitat.

**Figure 7.**
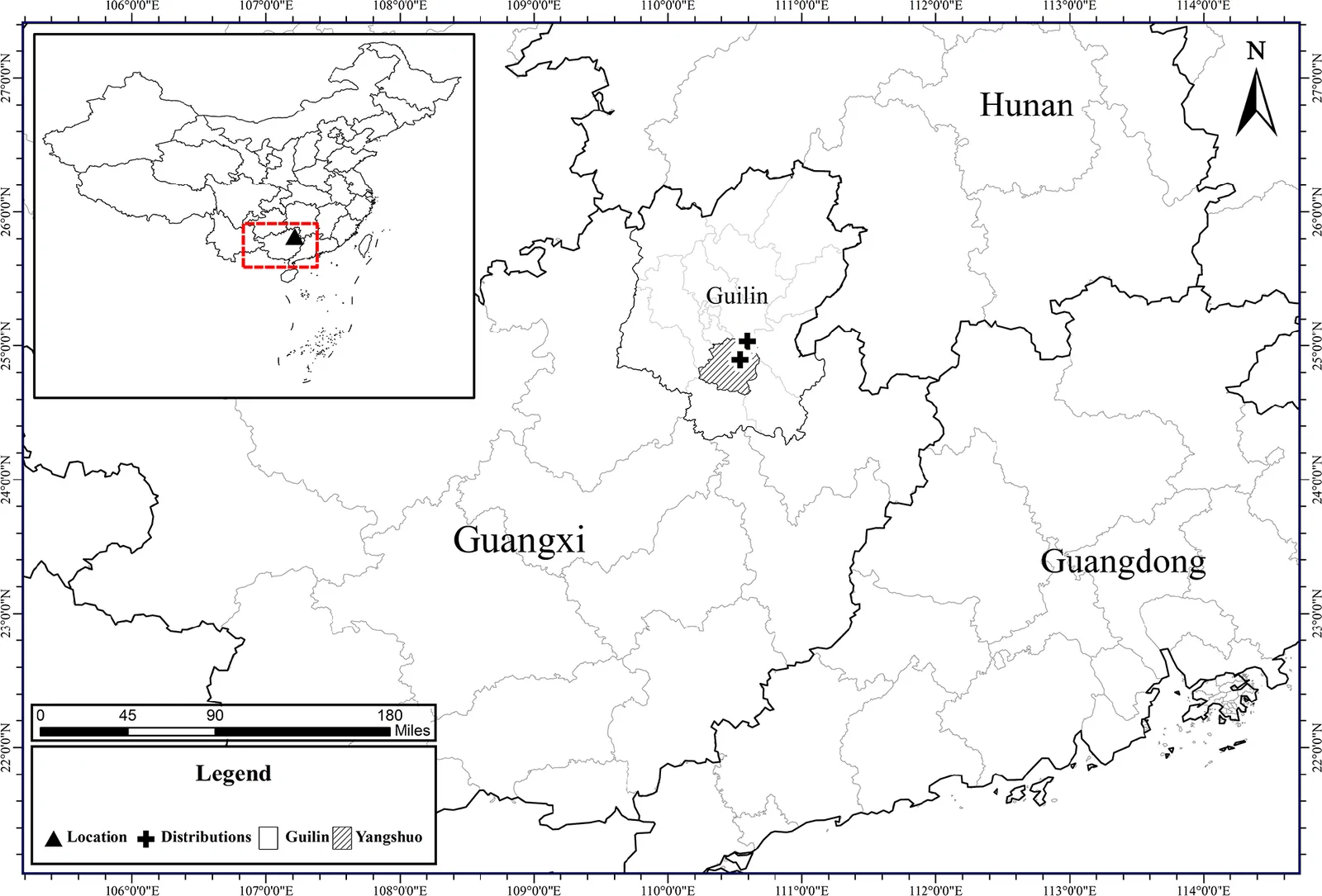
Distribution map of *Trichosanthes metamorpha*.

*Trichosanthes metamorpha* is currently known only from Shihuiyao and Chahualing in Yangshuo County (Fig. 7). It occurs mainly at 300–1200 m elevation, growing beneath damp rock walls and on forested slopes in relatively cool and moist environments. It is also found in cultivated fields in mountainous areas, where local farmers often regard it as a weed that negatively affects citrus cultivation. Rootstocks/rhizome fragments severed during hoeing and discarded along roadsides were observed to resprout and produce new climbing shoots. The habitat comprises a tree layer dominated by *Cunninghamia lanceolata* (Lamb.) Hook. and *Pinus* sp.; a shrub layer including *Boehmeria nivea* (L.) Gaudich., *Rubus alceifolius* Poir., and *Paliurus ramosissimus* (Lour.) Poir.; and an herb layer mainly consisting of *Nephrolepis cordifolia* (L.) C. Presl. Nymphs of the melon stink bug *Megymenum brevicorne* (Fabricius, 1787) were observed feeding on stems and leaves, although the extent of damage remains unclear. A pronounced heteroblastic change in leaf morphology was observed: during early growth, basal leaves are entire to shallowly lobed, whereas at maturity, apical leaves become deeply lobed with an increased number of lobes. The ecological or developmental drivers of these leaf-shape changes require further study. In addition, white fungal mycelia densely colonizing the leaf litter around the root zone were frequently observed in robust, flowering individuals. Male inflorescences strongly attract ants; ants were commonly observed beneath most male bracts. Male flowers open before dawn and emit an odor reminiscent of mushrooms and citrus. Female individuals appeared to be rare in the surveyed populations. Natural sources of mortality observed include fungal infection and root damage caused by pillbugs (Isopoda: Armadillidiidae).

##### Conservation status.

Two populations of *Trichosanthes metamorpha* were documented at Shihuiyao and Chahualing, each comprising more than 40 individuals. Twenty georeferenced records from these sites were analyzed in GeoCAT, giving an EOO of 20.288 km^2^ and an AOO of 40 km^2^ (2 × 2 km cells). The two sites are treated as two locations. Access to the surrounding mountain slopes is difficult, and the distribution outside these sites has not been adequately investigated; additional plants may therefore remain undetected. Roadside clearing practices (e.g., herbicide spraying) and plant harvesting are current anthropogenic threats and may reduce the number of mature individuals. Following the IUCN Red List Categories and Criteria and the Guidelines for Using the IUCN Red List Categories and Criteria (IUCN 2024), *T. metamorpha* is provisionally assessed as Vulnerable, VU B1ab(v)+2ab(v).

##### Etymology.

The specific epithet “metamorpha” refers to the stable and marked differences in leaf morphology between basal and apical leaves.

### A key to the species of *Trichosanthes* sect. *Involucraria* (Ser.) Wight in China

**Table:** 

1	Leaves palmately compound with 3–5 leaflets; involucral bracts rhomboid-lanceolate, coarsely toothed or sharply serrate in the middle to upper part	**2**
–	Leaves simple, 3–7 lobed; involucral bracts broadly ovate or oblong, concave, margin sharply toothed, rarely entire	**4**
2	Fruit ellipsoid; leaflets sessile, lanceolate, margin entire	**1. *T. quinquefolia***
–	Fruit globose; leaflets petiolulate, margin denticulate or sinuate-dentate	**3**
3	Tendrils 2-fid; stem slender, not densely lenticellate; fruit orange-yellow, apex without a protrusion	**2. *T. pedata***
–	Tendrils 3-fid; stem robust, densely lenticellate; fruit red, apex with a short protrusion (c. 2 mm)	**3. *T. napoensis***
4	Flowers white	**5**
–	Flowers red or pinkish	**11**
5	Involucral bracts entire	**6**
–	Involucral bract margins sharply toothed	**7**
6	Bracts ovate; leaf blade usually pentagonal, shallowly to moderately 5-lobed	**4. *T. quinquangulata***
–	Bracts oblong; leaf blade broadly ovate-cordate, entire or slightly triangular-lobed	**5. *T. cordata***
7	Involucral bracts oblong-lanceolate; bracteoles with white scaly hairs on both surfaces	**6. *T. tricuspidata***
–	Involucral bracts broadly elliptic; bracteoles without white scaly hairs on both surfaces	**8**
8	Leaf blade leathery (rarely membranous when young), usually 3–5 shallowly lobed, lobes triangular; bracts suborbicular, c. 4 cm in diam.; calyx lobes narrowly ovate, margins with 2–5 long acute lobes	**7. *T. lepiniana***
–	Leaf blade membranous or papery, entire or irregularly 2–3 shallowly to moderately lobed, or 3–7 deeply lobed; bracts ovate-saccate or broadly ovate, usually < 4 cm in diam.; calyx lobes lanceolate to linear, entire or toothed	**9**
9	Leaf lobes with undulate teeth or further shallowly lobed; bracteoles broadly ovate; adaxial surface densely prickly; abaxial surface prickly along veins; petiole prickly	**8. *T. laceribractea***
–	Leaf segments not further lobed; bracts ovate-saccate or broadly ovate; adaxial surface bristly or pubescent, abaxial surface glabrous; petiole glabrous or pubescent	**10**
10	Leaf blade cordate to broadly ovate-cordate, entire or 2–3-lobed to deeply lobed, lobes ovate-triangular; adaxial surface bristly; abaxial surface with dark spots near base; petiole glabrous; bracts ovate-saccate, margin irregularly lacerate; calyx lobes lanceolate, entire or toothed	**9. *T. fissibracteata***
–	Leaf blade suborbicular or broadly ovate, palmately 5–7-lobed, lobes oblong; adaxial surface pubescent; base without dark spots; petiole pubescent; bracts broadly ovate, middle to upper part with awl-shaped sharp teeth; calyx lobes linear, entire	**10. *T. wallichiana***
11	Stems, petioles, leaf surfaces, leaf veins, and tendrils all with punctations; leaf segments ovate-oblong; calyx lobes entire; seeds ovoid, inflated, pine nut-like	**11. *T. dunniana***
–	Entire plant without punctations except the leaf blades; leaf segments ovate-oblong; calyx lobes entire; seeds ovate-oblong, flattened	**12**
12	Male inflorescence peduncle thicker than stem; bracts hairy; sepals toothed or further divided (occasionally entire)	**13**
–	Male inflorescence peduncle thinner than or as thick as stem; bracts glabrous; sepals entire	**14**
13	Seeds oblong-elliptic, 12 × 4–5 mm, black or brownish black, usually with concave ornaments on both surfaces	**12. *T. pubera***
–	Seeds ovate-oblong, 15 × 7 mm, grayish green, with coarse, raised reticulate pattern on both surfaces	**13. *T. rugatisemina***
14	Leaves palmately 5–7-lobed, shallowly to moderately lobed, adaxially dark green; basal leaves without white spots; male inflorescence peduncle thinner than stem; bracts nearly entire, without glandular spots; corolla pink; petal fringes filiform, unfolded, 2–4 mm long, flat and flimsy	**14. *T. subrosea***
–	Leaves 3–5-lobed, adaxially dark glaucous green; basal leaves with white patterns; male inflorescence peduncle about as thick as stem; bracts with numerous sharply lobed teeth and dark glandular spots; corolla pinkish apricot; petal fringes dentate, incurled, 2.0–2.5 mm in length, brittle and thick, curling inward	**15. *T. metamorpha***

## Discussion

Based on morphological and molecular evidence, this study supports the recognition of *T. metamorpha* as a distinct species. Although it shares morphological similarities with *T. dunniana* and *T. pubera*, particularly in terms of inflorescence and leaf shape, *T. metamorpha* can be reliably differentiated by a set of unique diagnostic features, such as its glabrous stems with tiny black dots, white-patterned leaves, and incurled, dentate petal fringes. This combination of morphological traits does not overlap with that of closely related taxa, thereby providing sufficient evidence for the taxonomic delimitation of this species. The molecular analyses consistently placed *T. metamorpha* within *T.* sect. *Involucraria*. However, its precise sister relationship within the section remains uncertain, as different datasets recovered slightly different relationships with limited to moderate support.

Both datasets also recovered *T. dunniana* and *T. reticulinervis* as a strongly supported clade separated from the main sect. *Involucraria* lineage. This result agrees with Lin et al. (2025), who recovered sect. *Involucraria* as two distantly related groups, and is also consistent with the relatively weak support for the section in the earlier molecular analysis of de Boer and Thulin (2012). Despite this phylogenetic subdivision, sect. *Involucraria* remains morphologically recognizable under the traditional infrageneric framework used here. It is characterized by a combination of persistent probracts, medium to large and often glandular male bracts, petals fimbriate at least in the upper half, typically red mature fruits with dark green or greenish black pulp, and one-locular seeds that are flat or slightly swollen with an indistinct or only weakly developed margin. Nevertheless, because the deeper placement of the *T. dunniana*–*T. reticulinervis* clade differs between the cp-CDS and three-locus phylogenies, and the present taxon and nuclear-locus sampling is limited, a formal reassessment of sectional boundaries is beyond the scope of this study. Broader taxon sampling and additional independent nuclear data will be required to clarify these relationships and reassess the infrageneric classification of *Trichosanthes*.

Among the six sections of *Trichosanthes* represented in China, sect. *Involucraria* differs from sect. *Foliobracteola* mainly in its dark green pulp and weakly margined seeds (vs. white, greenish white, or pinkish pulp and distinctly margined seeds); from sect. *Cucumeroides* in the presence of persistent probracts and one-locular seeds (vs. probracts absent and seeds three-locular with two hollow lateral locules); from sect. *Trichosanthes* in its predominantly dioecious habit, larger male bracts, and dark green pulp (vs. monoecious habit, minute male bracts, and orange to red pulp); from sect. *Truncata* in its usually cordate leaf bases, larger male bracts, and dark green pulp (vs. truncate or rounded leaf bases, minute male bracts, and whitish pulp); and from sect. *Pseudovariifera* in the absence of a pseudo-ovary in male flowers and its dark green pulp (vs. a pseudo-ovary present and white pulp). These characters provide a practical morphological framework for distinguishing the Chinese species assigned to sect. *Involucraria* and therefore support the utility of the identification key provided here. *T. metamorpha* agrees with this sectional concept in its persistent probracts, relatively large glandular male bracts, fimbriate petals, and dark green pulp, but its orange-yellow mature fruits and strongly reduced petal fringes broaden the known morphological variation within the group.

The notably shortened, thickened, and brittle petal fringes of *T. metamorpha* represent one of its most distinctive floral characters. Male flowers open before dawn and emit a noticeable scent, suggesting a possible association with nocturnal pollination, as reported in other species of *Trichosanthes* (Duyfjes and Pruesapan 2004; de Boer et al. 2012; Lu et al. 2021). Long and elaborate petal fringes have been implicated in pollinator attraction in *Trichosanthes*, particularly in moth-pollinated species (de Boer et al. 2012; Ushimaru et al. 2023). Whether the strongly reduced and thickened fringes of *T. metamorpha* have a comparable function or are associated with differences in its pollination biology remains unknown and requires direct observations of floral visitors and pollination effectiveness.

## Supplementary Material

XML Treatment for Trichosanthes metamorpha

## Additional information

### Artificial Intelligence (AI) use

The authors accept full responsibility for the content of the manuscript, including the disclosure of any use of AI.

No AI tools were used in the preparation of this manuscript.

### Author ORCIDs

R.-Z. Wang https://orcid.org/0009-0006-8710-7493

Z.-H. Ran https://orcid.org/0009-0000-5213-6936

H. Schaefer https://orcid.org/0000-0001-7231-3987

S.-W. Wang https://orcid.org/0000-0002-8785-5065

N. Wei https://orcid.org/0000-0002-0313-3912

Q.-F. Wang https://orcid.org/0000-0001-9143-8849

### Data availability

All of the data that support the findings of this study are available in the main text or Supplementary Information.

## Supplementary materials

Supplementary material 1 List of accessions Data type xlsx Explanation note **table SS1**. List of accessions of plastomes used in this study. **table S2**. List of accessions used in the concatenated three-locus phylogenetic analysis. https://binary.pensoft.net/file/1801628This dataset is made available under the Open Database License (http://opendatacommons.org/licenses/odbl/1.0/). The Open Database License (ODbL) is a license agreement intended to allow users to freely share, modify, and use this Dataset while maintaining this same freedom for others, provided that the original source and author(s) are credited. Ruo-Zhou Wang, Zhao-Hui Ran, Hanno Schaefer, Sheng-Wei Wang, Neng Wei, Qing-Feng Wang

## References

[B1] Altschul SF, Gish W, Miller W, Myers EW, Lipman DJ (1990) Basic local alignment search tool. Journal of Molecular Biology 215(3): 403–410. 10.1016/S0022-2836(05)80360-2 2231712

[B2] Blume CL (1826) *Trichosanthes pubera* Blume. Bijdragen tot de Flora van Nederlandsch Indië 15: 936. 10.5962/bhl.title.6656

[B3] de Boer HJ, Thulin M (2012) Synopsis of *Trichosanthes* (Cucurbitaceae) based on recent molecular phylogenetic data. PhytoKeys 12: 23–33. 10.3897/phytokeys.12.2952 PMC3349053 22645411

[B4] de Boer HJ, Schaefer H, Thulin M, Renner SS (2012) Evolution and loss of longfringed petals: a case study using a dated phylogeny of the snake gourds (Cucurbitaceae). BMC Evolutionary Biology 12(1): 108. 10.1186/1471-2148-12-108 PMC3502538 22759528

[B5] de Wilde WJJO, Duyfjes BEE (2010) Cucurbitaceae. Flora Malesiana - Series 1. Spermatophyta 19(1): 1–333.

[B6] Duyfjes BEE, Pruesapan K (2004) The genus *Trichosanthes* L. (Cucurbitaceae) in Thailand. Thai Forest Bulletin (Botany) 32: 76–109.

[B7] Farris JS, Källersjö M, Kluge AG, Bult C (1995) Constructing a significance test for incongruence. Systematic Biology 44(4): 570–572. 10.1093/sysbio/44.4.570

[B8] Hooker JD, Thomson T (1854) On *Hodgsonia*, Hook. fil. et Thomas., a new and remarkable genus of Cucurbitaceae. Proceedings of the Linnean Society of London 2: 257–259.

[B9] Huang LQ, Jeffrey C (2011) *Trichosanthes*. In: Wu CY, Raven P, Hong DY (Eds) Flora of China. Vol. 19. Science Press, Beijing & Missouri Botanical Garden Press, St Louis, 1–37.

[B10] Huang LQ, Yang B, Yue CX, Cheng CY (1997) Pollen morphology of *Trichosanthes* and its taxonomic significance. Acta Phytotaxonomica Sinica 35(2): 125–135.

[B11] IUCN (2024) Guidelines for using the IUCN Red List Categories and Criteria. Version 16. Prepared by the Standards and Petitions Committee of the IUCN Species Survival Commission. https://www.iucnredlist.org/resources/redlistguidelines

[B12] Jin JJ, Yu WB, Yang JB, Song Y, de Pamphilis CW, Yi TS, Li DZ (2020) GetOrganelle: a fast and versatile toolkit for accurate *de novo* assembly of organelle genomes. Genome Biology 21: 241. 10.1186/s13059-020-02154-5 PMC7488116 32912315

[B13] Katoh K, Standley DM (2013) MAFFT multiple sequence alignment software version 7: improvements in performance and usability. Molecular Biology and Evolution 30(4): 772–780. 10.1093/molbev/mst010 PMC3603318 23329690

[B14] Kocyan A, Zhang LB, Schaefer H, Renner SS (2007) A multi-locus chloroplast phylogeny for the Cucurbitaceae and its implications for character evolution and classification. Molecular Phylogenetics and Evolution 44(2): 553–577. 10.1016/j.ympev.2006.12.022 17321763

[B15] Lanfear R, Frandsen PB, Wright AM, Senfeld T, Calcot B (2017) PartitionFinder 2: new methods for selecting partitioned models of evolution. Molecular Biology and Evolution 34(3): 772–773. 10.1093/molbev/msw260 28013191

[B16] Letunic I, Bork P (2021) Interactive Tree of Life (iTOL) v5: an online tool for tree display and annotation. Nucleic Acids Research 49(W1): W293–W296. 10.1093/nar/gkab301 PMC8265157 33885785

[B17] Léveillé AH (1911) *Trichosanthes dunniana* H. Lév. Repertorium Specierum Novarum Regni Vegetabilis 10: 148. 10.1002/fedr.19110101002

[B18] Lin DL, Wen YY, Wang ZQ, Zhang DL, Li ZM, Liu H, Chen LX, Li LS, Yang JY, Yang WC (2025) Elucidation of the phylogeny of Cucurbitaceae, particularly *Trichosanthes*, based on plastome data and nuclear single-copy genes. BMC Plant Biology 25: 929. 10.1186/s12870-025-06970-4 PMC12275332 40684091

[B19] Linnaeus C (1753) Species Plantarum. Laurentius Salvius, Stockholm, 1200 pp.

[B20] Liu Q, Lin N, Zhang DG, Huang XH, Wang HC, Yang JY, Tojibaev K, Lv ZY, Deng T, Li ZM (2021) *Trichosanthes sunhangii* (Cucurbitaceae), a new species from Hubei, China. Phytotaxa 479(3): 287–294. 10.11646/phytotaxa.479.3.5

[B21] Lu QB, Liu CQ, Huang SX (2021) Moths pollinate four crops of Cucurbitaceae in Asia. Journal of Applied Entomology 145(6): 499–507. 10.1111/jen.12871

[B22] Maximowicz CJ (1859) *Trichosanthes kirilowii* Maxim. Primitiae Florae Amurensis, 482 pp.

[B23] Miller P (1754) *Luffa* Mill. Gardener’s Dictionary, Abridged, 4^th^ edn.

[B24] Nguyen LT, Schmidt HA, von Haeseler A, Minh BQ (2015) IQ-TREE: a fast and effective stochastic algorithm for ML phylogeny inference. Molecular Biology and Evolution 32(1): 268–274. 10.1093/molbev/msu300 PMC4271533 25371430

[B25] Nong DX, Huang BY, Lan ZZ, Xie DM, Yu LY, Huang LQ (2015) *Trichosanthes napoensis* (Cucurbitaceae), a new species from Guangxi, China. Phytotaxa 207(3): 297–300. 10.11646/phytotaxa.207.3.9

[B26] Ronquist F, Teslenko M, van der Mark P, Ayres DL, Darling A, Höhna S, Larget B, Liu L, Suchard MA, Huelsenbeck JP (2012) MrBayes 3.2: efficient Bayesian phylogenetic inference and model choice. Systematic Biology 61(3): 539–542. 10.1093/sysbio/sys029 PMC3329765 22357727

[B27] Rugayah R, de Wilde WJJO (1999) Conspectus of *Trichosanthes* (Cucurbitaceae) in Malesia. Reinwardtia 11(4): 227–280.

[B28] Swofford DL (2002) PAUP. Phylogenetic Analysis Using Parsimony (and other methods). Version 4.0b10. Sinauer Associates, Sunderland, MA, USA.

[B29] Thiers B (2026) Index Herbariorum: A global directory of public herbaria and associated staff. New York Botanical Garden’s Virtual Herbarium. http://sweetgum.nybg.org/science/ih/ [Accessed 08.04.2026]

[B30] Tillich M, Lehwark P, Pellizzer T, Ulbricht-Jones ES, Fischer A, Bock R, Greiner S (2017) GeSeq – versatile and accurate annotation of organelle genomes. Nucleic Acids Research 45(W1): W6–W11. 10.1093/nar/gkx391 PMC5570176 28486635

[B31] Ushimaru A, Seo N, Sakagami K, Funamoto D (2023) Sexual dimorphism in specialist pollinated dioecious species with complex flowers: *Trichosanthes cucumeroides* and the role of petal fringes in hawkmoth attraction. American Journal of Botany 110(5): 1120–1133. 10.1002/ajb2.16148 36852530

[B32] Yueh CH, Cheng CY (1980) The Chinese medicinal species of the genus *Trichosanthes* L. Journal of Systematics and Evolution 18(3): 333–352.

